# The Prognostic Significance of the Systemic Inflammation Response Index (SIRI) and HALP Score in Hodgkin’s Lymphoma

**DOI:** 10.3390/diagnostics16070980

**Published:** 2026-03-25

**Authors:** Kübra Oral, Ayşe Uysal

**Affiliations:** Department of Hematology, Faculty of Medicine, Firat University, Elazığ 23300, Turkey; auysal@firat.edu.tr

**Keywords:** Hodgkin’s lymphoma, systemic inflammation response index (SIRI), HALP score

## Abstract

**Objective**: This study aimed to evaluate the prognostic significance of lymphocyte-associated inflammatory markers and the HALP score in patients with Hodgkin’s lymphoma. **Methods**: This was a retrospective study that included patients who were diagnosed with Hodgkin’s lymphoma and followed up between 2004 and 2024. The inflammatory markers (NLR, PLR, MLR, SII, SIRI, and PIV) and HALP score were calculated from the patients’ biochemical and hematological parameters, and the relationship between these parameters and stage, spleen and liver involvement, relapse, mortality, overall survival, and progression-free survival was analyzed. **Results**: A total of 117 patients were included, and multivariate analysis indicated that progression-free survival was statistically and significantly associated with treatment type (*p* = 0.0285), PLR (*p* = 0.0188), and PIV (*p* = 0.0297). In terms of overall survival, age (*p* = 0.0011), treatment type (*p* = 0.0108), and SIRI (*p* = 0.0108) remained as statistically significant predictors. Although the HALP score showed a significant association with PFS in the univariate analysis (*p* = 0.0104), this association did not persist in the multivariate model. In addition, no statistically significant relationship between the HALP score and OS was observed in either the univariate or multivariate analysis. **Conclusions**: The SIRI is a prognostic marker in Hodgkin’s lymphoma and may be useful for predicting overall survival.

## 1. Introduction

Hodgkin’s lymphoma (HL) is a neoplasm originating from the lymphatic system, and it is classified into two distinct types: classical Hodgkin’s lymphoma (cHL) and nodular lymphocyte-predominant Hodgkin’s lymphoma (NLPHL). Although the tumor cells in both classes originate from germinal center B cells, notable morphological and phenotypic differences are observed between the two classifications. Classical Hodgkin lymphoma (HL) is characterized by the presence of Hodgkin and Reed–Sternberg (HRS) cells. Despite the abundant inflammatory infiltrate within the tumor microenvironment, HRS cells typically constitute less than 1% of the total cellular composition of the tumor. This finding indicates that tumor cells in HL develop due to widespread inflammation [1]. Furthermore, tumor-associated macrophages and neutrophils suppress the cytotoxic T-cell response by activating immune checkpoint pathways through the release of immunosuppressive cytokines and chemokines, thereby contributing to tumor progression [2].

The availability of prognostic indices as a guide for patient treatment is of great importance. In the context of classical HL, prognostic factors based on staging according to the Ann Arbor classification indicate that an elevated erythrocyte sedimentation rate (ESR) is utilized as a negative risk factor in patients with early-stage disease (Stages 1–2) [3]. Conversely, laboratory parameters such as leukocytosis, anemia, lymphopenia, and low albumin levels are employed for the prognosis of patients with advanced-stage disease (Stages 3–4) [4].

Recent studies have demonstrated that systemic inflammation plays a significant role in cancer progression, patient survival, and chemotherapy response [5]. Moreover, anemia and malnutrition are significant contributors to tumor prognosis [6]. In light of the available data, there is mounting recognition that several biomarkers are vital for predicting cancer prognosis in patients. These biomarkers include the neutrophil–lymphocyte ratio (NLR), the platelet–lymphocyte ratio (PLR), the monocyte–lymphocyte ratio (MLR), the systemic immune–inflammation index (SII), the systemic inflammation response index (SIRI), the pan-immune–inflammation value (PIV), and the hemoglobin, albumin, lymphocyte, and platelet (HALP) score [7,8,9]. The HALP score provides a comprehensive assessment of a patient’s nutritional status in addition to evaluating their inflammatory response [10,11,12].

This study aimed to determine the prognostic significance of the HALP and lymphocyte-related inflammatory parameters (NLR, PLR, MLR, SII, SIRI, and PIV) that have been previously employed for prognosis in solid tumors, specifically in cHL.

## 2. Materials and Methods

### 2.1. Study Design and Patient Selection

This retrospective study included 117 patients diagnosed with Hodgkin’s lymphoma at the Adult Hematology Clinic of the Firat University Faculty of Medicine between November 2004 and October 2024. Ethics committee approval was obtained from the Fırat University Non-Interventional Scientific Research Ethics Committee (approval no: 2025/15-14; date: 23 October 2025). Demographic, clinical, and laboratory data at the time of diagnosis were retrospectively reviewed, with patient data obtained from their files and the institution’s hospital information system. This study’s inclusion criteria were as follows: (a) patients pathologically diagnosed with classical Hodgkin’s lymphoma, (b) patients over the age of 18, and (c) patients with blood samples available prior to treatment administration. The exclusion criteria were as follows: (a) missing data, (b) active infection, (c) secondary malignancy, and (d) autoimmune disease.

### 2.2. Data Collection

The following variables were the primary focus of this study: patient age, sex, survival status, presence of relapsed disease, follow-up time, disease stage according to the Ann Arbor staging system, histological subtype, liver and spleen involvement, treatment type, laboratory parameters (hemoglobin, neutrophil, monocyte, lymphocyte, platelet, albumin, lactate dehydrogenase (LDH), erythrocyte sedimentation rate (ESR), and C-reactive protein (CRP) levels), and overall and progression-free survival of patients. Overall survival (OS) was defined as the time from diagnosis to death or the last follow-up date, while progression-free survival (PFS) was defined as the time from the remission date after treatment until relapse, the last follow-up date, or death. Primary refractory patients were not included in the PFS calculation. The most recent follow-up date was 30 June 2025.

Liver and spleen involvement was defined according to the following criteria:

• Increased FDG uptake consistent with lymphoma in the affected organ on PET/CT imaging (focal or diffuse, consistent with clinical and other imaging findings);
• Presence of parenchymal lesions suggestive of lymphomatous infiltration on CT or PET/CT imaging;
• Isolated organomegaly alone was not considered sufficient; organ enlargement was only evaluated as lymphomatous involvement if it was accompanied by increased metabolic activity or radiological findings of infiltrative involvement.

A comprehensive evaluation of the patients was conducted, encompassing various analytical metrics. These included the neutrophil-to-lymphocyte ratio (NLR), the platelet-to-lymphocyte ratio (PLR), the monocyte-to-lymphocyte ratio (MLR), the systemic immune–inflammation index (SII), the systemic inflammation response index (SIRI), the pan-immune–inflammation value (PIV), and the HALP score. Calculations were performed as described in the following section.

☐ HALP: Hemoglobin (g/L) × albumin (g/L) × lymphocyte (/L)/platelet (/L) [13];
☐ SII: Platelet (10^3^/µL) × neutrophil (10^3^/µL)/lymphocyte (10^3^/µL) [14];
☐ SIRI: Neutrophil (10^3^/µL) × monocyte (10^3^/µL)/lymphocyte (10^3^/µL) [14];
☐ PIV: Platelet (10^3^/µL) × neutrophil (10^3^/µL) ×monocyte (10^3^/µL)/lymphocyte (10^3^/µL) [15].

The cut-off values for the NLR, PLR, MLR, SII, SIRI, PIV, and HALP were 4.33, 304.82, 0.602, 1127.97, 2.307, 433.92, and 21.93, respectively. (The table was provided as a Supplementary File).

### 2.3. Statistical Analysis

Statistical analyses were conducted using IBM SPSS Statistics for Windows, Version 23.0 (Statistical Package for the Social Sciences, IBM Corp., Armonk, NY, USA). Descriptive statistics were presented as *n* and % for categorical variables and the median (min–max) for continuous variables. ROC curve analysis was performed on the various indices to predict mortality. The cut-off values were calculated for meaningful continuous data only, and the means of the maximum specificity and sensitivity were selected as a cut-off values. These values were derived using ROC analysis with Youden index and given in Digital Supplementary File. Pearson’s chi-squared test and Fisher’s exact test were employed to compare categorical variables, while the Kaplan–Meier method was employed to compare the overall and progression-free survival times between the clinical groups. Statistically significant data based on the univariate analysis were screened for multiple correlations before being included in the multivariate analysis to prevent multicollinearity. Variance Inflation Factor (VIF) > 5 was used for exclusion from the multivariate analysis. Multivariable Cox regression was used at last stage. *p* < 0.05 was selected for the statistical significance of the alpha. The Type 1 error of the multiple univariate comparisons were adjusted via the Benjamini–Hochberg FDR formula.

## 3. Results

As shown in Table 1, this study included a total of 117 patients. The median age of the patients was 39 years (range, 18–88 years). The population was predominantly male (63.2% male (*n* = 74); 36.8% female (*n* = 43)). Fifty-nine percent of patients were in the advanced stage (*n* = 69), while 41 percent were in the early stage (*n* = 48). The prevalence of liver involvement was 27.4% among the patient population (*n* = 32), while that of splenic involvement was 35.9% (*n* = 42). Among the histopathological subtypes, the most prevalent was nodular sclerosing, accounting for 45.3% of cases (*n* = 53). In contrast, lymphocyte-depleted Hodgkin’s lymphoma was the least common form, with a prevalence of 3.4% (*n* = 4). Most patients, 88.9% (*n*: 104), were receiving ABVD treatment. Specifically, twelve patients had received brentuximab-based treatment, while only one patient was monitored without treatment. The median albumin, CRP, LDH, ESR levels were 41 g/L, 22 mg/L, 239 u/L, and 40 mm/h, respectively, and 74 patients had an ESR > 30 mm/h.

The relationships between the NLR, the PLR, and the MLR and age, gender, treatment type, stage, liver and spleen involvement, mortality, relapse, PFS, OS, and IPS were evaluated. Statistically significant correlations were found for MLR with spleen involvement (*p* = 0.015), PFS (*p* = 0.0038), and OS (*p* = 0.0025). The relationship between the NLR, the PLR, and the MLR and clinical findings is shown in Table 2.

The relationships between SII, SIRI, PIV, HALP score and age, gender, treatment type, stage, liver and spleen involvement, mortality, relapse, PFS, OS, and IPS were evaluated. SIRI showed statistically significant differences in mortality (*p* = 0.033), PFS (*p* = 0.0077), and OS (*p* = 0.0462); PIV showed statistically significant differences in PFS (*p* = 0.0069), and OS (*p* = 0.0128), and the HALP score showed statistically significant differences stage (*p* = 0.042). The relationship between SII, SIRI, PIV, the HALP score and clinical findings is shown in Table 3.

In the total cohorts, at the median 62 months follow-up, median PFS and OS were not reached (NR) (95% Confidence interval (CI) NR-NR).

Clinical and inflammatory parameters were analyzed using univariate analysis with PFS. According to the univariate analysis, treatment type (*p* = 0.0305), stage (*p* = 0.0212), ESR (*p* = 0.0190), the NLR (*p* = 0.0040), the PLR (*p* = 0.0041), the MLR (*p* = 0.0017), SII (*p* = 0.0116), SIRI (*p* = 0.0063), PIV (*p* = 0.0016), and HALP (*p* = 0.0104) were statistically significant. When multivariate analysis was performed with the parameters that were significant in the univariate analysis, treatment type (*p* = 0.0285), the PLR (*p* = 0.0188), and PIV (*p* = 0.0297) were found to be statistically significant. The PFS univariate and multivariate results are shown in Table 4.

Clinical and inflammatory parameters were examined using univariate analysis with OS. According to univariate analysis, age (*p* = 0.0001), being over 65 years old (*p* = 0.0001), treatment type (*p* = 0.0001), an ESR above 30 mm/h (*p* = 0.0123), the NLR (*p* = 0.0228), the MLR (*p* = 0.0009), SIRI (*p* = 0.0004), and PIV (*p* = 0.0018) were found to be statistically significant. Multivariate analysis was performed on the parameters that were statistically significant in the univariate analysis, and age (*p* = 0.0011), treatment type (*p* = 0.0108), and SIRI (*p* = 0.0108) were found to be statistically significant. The OS univariate and multivariate results are shown in Table 5.

## 4. Discussion

Hodgkin’s lymphoma is a neoplasm that originates from lymphocytes in the lymphoid tissues. Recent advancements in treatment outcomes, marked by a decline in long-term adverse effects, have been achieved by incorporating negative risk factors and response-based treatment methodologies. Prognostic factors function as guidelines that facilitate an effective treatment–toxicity balance in patients. In early-stage patients (stages 1–2), B symptoms, mediastinal mass, bulky disease, and elevated ESR are considered adverse risk factors. In contrast, in advanced-stage patients (stages 3–4), prognostic factors such as hypoalbuminemia, lymphopenia, anemia, and leukocytosis are utilized. This study aimed to evaluate the effects of the NLR, PLR, MLR, SII, SIRI, PIV, and HALP scores on Hodgkin’s lymphoma. These scores have been previously investigated as prognostic markers in several cancer types.

In the present study, we retrospectively examined 117 patients diagnosed with Hodgkin’s lymphoma to assess the correlation between inflammatory parameters and disease stage, liver and spleen involvement, relapse, mortality, overall survival, and progression-free survival. The findings of this study indicate that although the NLR, PLR, MLR, SII, SIRI, PIV, and HALP scores were statistically significant for PFS according to the cut-off values in the univariate analysis, only PLR and PIV remained statistically significant in the multivariate analysis. In contrast, NLR, MLR, SIRI, and PIV were significantly associated with OS in the univariate analysis; however, only SIRI remained statistically significant in the multivariate analysis.

Research has demonstrated that elevated SIRI levels are associated with poor prognosis in solid cancers, including those of the stomach [16], liver [17], and pancreas [12]. Despite the plethora of studies in the extant literature addressing the impact of inflammation parameters on the prognosis of solid organ cancers, the relationship between inflammation and lymphomas remains underexplored, as most studies have focused on non- Hodgkin’s lymphomas (NHLs). Matsuda et al. investigated the association between several biomarkers and survival outcomes in 47 patients diagnosed with primary central nervous system (CNS) lymphoma. Their findings revealed that the lymphocyte-to-monocyte ratio (LMR), the NLR, and the PLR did not significantly affect overall survival (OS). However, high SII and SIRI scores were identified as independent prognostic factors associated with an elevated mortality risk [18]. In a separate study on primary central nervous system lymphoma (PCNSL), Feng et al. evaluated 73 patients with PCNSL. They found that high NLR, PLR, SII, and SIRI scores were associated with shorter OS and PFS [19]. In a separate study of an NHL subgroup, Chu et al. interpreted the NLR, LMR, SII, and SIRI as significant variables for OS and PFS in their study of 153 patients with primary gastrointestinal diffuse large B-cell lymphoma (DLBCL) [20]. To our knowledge, studies specifically evaluating SIRI as a prognostic factor in Hodgkin’s Lymphoma remain scarce.

Our findings suggest a potential association between elevated SIRI values and poorer OS outcomes in patients with HL; however, these results are exploratory and should be validated in future studies.

The HALP score is a simple marker used in clinical practice, providing insights into the nutritional and immune status of patients. The term was initially introduced by Chen et al. in 2015 to predict gastric carcinoma prognosis in patients [13]. The investigation revealed that participants with elevated HALP scores demonstrated superior OS outcomes [13]. Subsequently, the relationship between OS and HALP scores was examined in solid cancers, including pancreatic [21], bile duct [22], small cell lung [23], and bladder [24] cancers. The results indicated that overall survival was superior in the groups with higher HALP scores. In a study involving 153 DLBCL patients, Vlatka et al. demonstrated that, based on the determined cut-off value of 20.8 for the HALP score, patients with low HALP scores presented with more advanced disease and had significantly poorer survival [25]. Similarly, the research conducted by Çetintepe et al. in their study on DBBHL revealed that patients with low HALP scores (a cut-off point of 26.17, as determined by the study’s 201 patient sample) demonstrated more advanced disease stages and poorer survival outcomes [26]. Additionally, the same conclusion was obtained in the same disease group despite variations in sample size and cut-off thresholds. The only study in the literature examining the relationship between Hodgkin’s lymphoma and HALP scores, where patients with classic Hodgkin’s lymphoma only were included, found no statistically significant results in survival for patients with low HALP scores based on a cut-off point of 19.9. The aforementioned study determined that elevated HALP scores in the early stage, along with diminished NLR and PLR scores and heightened LMR scores, were statistically significant [27]. In our study of 117 patients, lower HALP scores were observed in patients with advanced-stage disease, as defined by a cut-off value of 21.93. This finding is consistent with the results of previously published studies. Although studies on non-Hodgkin’s lymphoma have demonstrated a significant correlation between HALP scores and survival, no significant association with survival was observed in our cohort, similar to the only Hodgkin’s lymphoma study conducted in the literature.

## 5. Limitation

The limitations of this study stem from its modest sample size, restriction to a single center, and retrospective nature. Despite its classification as a singular disease entity, Hodgkin’s lymphoma encompasses a heterogeneous group of diseases due to the presence of different subtypes. Additionally, the heterogeneity of the population may have impeded the generalizability of the results. Consequently, prospective studies with larger patient numbers and more homogeneous patient populations, where each subgroup is evaluated individually, are necessary. The limitations of our study also include the inability to assess B symptoms, bulky disease, and extranodal involvement of organs other than the liver and spleen.

## 6. Conclusions

Although this study is limited by its retrospective and single-center design, the relatively long median follow-up period (62 months) offers a comprehensive real-world perspective on Hodgkin’s lymphoma. Although HALP scores were statistically significant in advanced stage, splenic involvement, and relapse, no significant results were obtained in survival data.

The SIRI threshold value proposed in our study was considered for exploratory and hypothesis-forming purposes rather than for clinical use. We believe that validation in independent cohort and prospective studies is necessary before any clinical application can be recommended.

Thus, the SIRI may be developed as a reproducible, inexpensive, and easily accessible biomarker that can assist clinicians in assessing the prognosis and risk stratification of patients with Hodgkin’s lymphoma.

## Figures and Tables

**Table 1:** Patient demographic and clinical characteristics (*n* = 117).

Variables	*N*	%
Age		
Median (min–max)	39 (18–88)	
≤65	104	88.9
>65	13	11.1
Gender		
Female	43	36.8
Male	74	63.2
Stage		
Early Stage	48	41
Advanced Stage	69	59
Liver Involvement		
No	85	72.6
Yes	32	27.4
Spleen Involvement		
No	75	64.1
Yes	42	35.9
Subtype		
Nodular Sclerosis	53	45.3
Mixed Cellularity	37	31.6
Lymphocyte-rich	9	7.7
Lymphocyte-depleted	4	3.4
Classic Hodgkin’s (subtype not specified)	14	12
Treatment Type		
ABVD	104	88.8
BR-based	12	10.3
Other	1	0.9
Albumin		
Median (min–max)	41 (24–51)	
CRP		
Median (min–max)	22 (1–212)	
ESR		
Median (min–max)	40 (2–133)	
≤30	43	36.8
>30	74	63.2
LDH		
Median (min–max)	239 (120–815)	

**Table 2:** The baseline characteristics of the patients stratified according to the NLR, PLR, and MLR score groups.

			NLR ≥ 4.33	NLR < 4.33	*p*-Value *	PLR > 304.82	PLR ≤ 304.82	*p*-Value *	MLR > 0.602	MLR ≤ 0.602	*p*-Value *
No. Count	N		48	69	117	36	81	117	36	81	117
	%		41	59		30.8	69.2		30.8	69.2	
Age	Median		39	39	0.975	43	39	0.586	50	37	0.068
	Minimum		18	18		18	18		18	18	
	Maximum		84	88		84	88		88	80	
	≤65	N	42	62	0.983	31	73	0.664	29	75	0.204
		%	87.5	89.9		86.1	90.1		80.6	92.6	
	>65	N	6	7		5	8		7	6	
		%	12.5	10.1		13.9	9.9		19.4	7.4	
Gender	Male	N	25	49	0.127	19	55	0.297	26	48	0.386
		%	52.1	71		52.8	67.9		72.2	59.3	
	Female	N	23	20		17	26		10	33	
		%	47.9	29		47.2	32.1		27.8	40.7	
Treatment Type	ABVD	N	43	61	>0.999	33	71	0.876	27	77	0.051
		%	89.6	88.4		91.7	87.7		75	95.1	
	BR-based	N	5	7		3	9		8	4	
		%	10.4	10.1		8.3	11.1		22.2	4.9	
	Other	N	0	1		0	1		1	0	
		%	0	1.4		0	1.2		2.8	0	
Stage	Early	N	13	35	0.0693	9	39	0.091	11	37	0.306
		%	27.1	50.7		25	48.1		30.6	45.7	
	Advanced	N	35	34		27	42		25	44	
		%	72.9	49.3		75	51.9		69.4	54.3	
Liver Involvement	Yes	N	19	13	>0.999	15	17	0.099	11	21	0.882
		%	39.6	18.8		41.7	21		30.6	25.9	
	No	N	29	56		21	64		25	60	
		%	60.4	81.2		58.3	79		69.4	74.1	
Spleen Involvement	Yes	N	21	21	0.314	20	22	0.057	21	21	0.015
		%	43.8	30.4		55.6	27.2		58.3	25.9	
	No	N	27	48		16	59		15	60	
		%	53.6	69.6		44.4	72.8		41.7	74.1	
Mortality	Exitus	N	12	7	0.122	9	10	0.266	11	8	0.057
		%	25	10.1		25	12.3		30.6	9.9	
	Alive	N	36	62		27	71		25	73	
		%	75	89.9		75	87.7		69.4	90.1	
Relapse	Yes	N	18	11	0.067	15	14	0.057	14	15	0.095
		%	37.5	15.9		41.7	17.3		38.9	18.5	
	No	N	30	58		21	67		22	66	
		%	62.5	84.1		58.3	82.7		61.1	81.5	
PFS	Median		37	62	0.059	33	58	0.126	24	69	0.0038
	Minimum		2	1		2	1		1	2	
	Maximum		219	247		219	247		179	247	
OS	Median		53	83	0.205	53	69	0.576	40	83	0.0025
	Minimum		2	1		2	1		1	7	
	Maximum		219	247		219	247		179	247	
IPS	Low	N	15	25	NA	13	27	NA	10	30	NA
		%	37.5	62.5		32.5	67.5		25	75	
	Intermediate	N	16	6		9	13		10	12	
		%	72.7	27.3		40.9	59.1		45.5	54.5	
	High	N	4	3		5	2		5	2	
		%	57.1	42.9		71.4	28.6		71.4	28.6	

* Benjamini–Hochberg FDR correction was applied for multiple comparison.

**Table 3:** The baseline characteristics of the patients stratified according to the SII, SIRI, PIV and HALP score groups.

			SII > 1127.97	SII ≤ 1127.97	*p*-Value *	SIRI > 2.307	SIRI ≤ 2.307	*p*-Value *	PIV > 433.92	PIV ≤ 433.92	*p*-Value *	HALP < 21.93	HALP ≥ 21.93	*p*-Value *
No. Count	N		55	62	117	60	57	117	74	43	117	58	59	117
	%		47	53		51.3	48.7		63.2	36.8		49.6	50.4	
Age	Median		37	41	0.858	40	39	0.484	40	39	0.450	39	39	0.854
	Minimum		18	18		18	18		18	18		18	18	
	Maximum		84	88		88	70		88	70		84	88	
	≤65	N	48	56	0.912	50	54	0.193	64	40	0.497	50	54	0.674
		%	87.3	90.3		83.3	94.7		86.5	93		86.2	91.5	
	>65	N	7	6		10	3		10	3		8	5	
		%	12.7	9.7		16.7	5.3		13.5	7		13.8	8.5	
Gender	Male	N	28	46	0.0641	35	39	0.477	46	28	0.993	30	44	0.068
		%	50.9	74.2		58.3	68.4		62.2	65.1		51.7	74.6	
	Female	N	27	16		25	18		28	15		28	15	
		%	49.1	25.8		41.7	31.6		37.8	34.9		48.3	25.4	
Treatment Type	ABVD	N	50	54	0.983	50	54	0.262	63	41	0.315	52	52	0.914
		%	90.9	87.1		83.3	94.7		85.1	95.3		89.7	88.1	
	BR-based	N	5	7		9	3		10	2		5	7	
		%	9.1	11.3		15	5.3		13.5	4.7		8.6	11.9	
	Other	N	0	1		1	0		1	0		1	0	
		%	0	1.6		1.7	0		1.4	0		1.7	0	
Stage	Early	N	16	32	0.077	19	29	0.129	25	23	0.124	16	32	0.042
		%	29.1	51.6		31.7	50.9		33.8	53.5		27.6	54.2	
	Advanced	N	39	30		41	28		49	20		42	27	
		%	70.9	48.4		68.3	49.1		66.2	46.5		72.4	45.8	
Liver Involvement	Yes	N	19	13	0.262	18	14	0.796	20	12	>0.999	21	11	0.126
		%	34.5	21		30	24.6		27	27.9		36.2	18.6	
	No	N	36	49		42	43		54	31		37	48	
		%	65.5	79		70	75.4		73	72.1		63.8	81.4	
Spleen Involvement	Yes	N	23	19	0.424	27	15	0.055	29	13	0.582	28	14	0.055
		%	41.8	30.6		45	26.3		39.2	30.2		48.3	23.7	
	No	N	32	43		33	42		45	30		30	45	
		%	58.2	69.4		55	73.7		60.8	69.8		51.7	76.3	
Mortality	Exitus	N	12	7	0.316	16	3	0.033	17	2	0.069	12	7	0.430
		%	21.8	11.3		26.7	5.3		23	4.7		20.7	11.9	
	Alive	N	43	55		44	54		57	41		46	52	
		%	78.2	88.7		73.3	94.7		77	95.3		79.3	88.1	
Relapse	Yes	N	19	10	0.095	20	9	0.120	24	5	0.073	20	9	0.089
		%	34.5	16.1		33.3	15.8		32.4	11.6		34.5	15.3	
	No	N	36	52		40	48		50	38		38	50	
		%	65.5	83.9		66.7	84.2		67.6	88.4		65.5	84.7	
PFS	Median		41	66	0.092	36	74	0.0077	37	76	0.0069	44	58	0.206
	Minimum		2	1		1	3		1	5		1	7	
	Maximum		219	247		219	247		219	247		219	247	
OS	Median		53	79	0.306	49	90	0.0462	49	94	0.0128	62	62	>0.999
	Minimum		2	1		1	9		1	9		1	8	
	Maximum		219	247		219	247		219	247		219	247	
IPS	Low	N	18	22	NA	20	20	NA	26	14	NA	19	21	NA
		%	45	55		50	50		65	35		47.5	52.5	
	Intermediate	N	17	5		17	5		19	3		16	6	
		%	77.3	22.7		77.3	22.7		86.4	13.6		72.7	27.3	
	High	N	4	3		4	3		4	3		7	0	
		%	57.1	42.9		57.1	42.9		51.7	42.9		100	0	

* Benjamini–Hochberg FDR correction was applied for multiple comparison.

**Table 4:** Univariate and multivariate analyses of progression-free survival.

	Univariate Analysis				Multivariate Analysis		
Parameters For PFS	Hazard Ratio	95% Confidence Interval	*p*	VIF	Hazard Ratio	95% Confidence Interval	*p*
Age (years)	1.0158	0.9943–1.0376	0.1534	-			
Age (>65 years)	0.8924	0.2104–3.7844	0.8754	-			
Sex (male/female)	0.6955	0.3164–1.5285	0.3561	-			
Treatment Type (ABVD vs. BV-based)	0.2839	0.1029–0.7833	0.0305	1.2	0.3155	0.1124–0.8858	0.0285
Stage	0.3923	0.1673–0.9195	0.0212	1.2	-	-	-
Liver involvement	1.4114	0.6558–3.0373	0.3875	-			
Spleen involvement	1.4714	0.7011–3.0878	0.3137	-			
ESR	1.0136	1.0025–1.0247	0.0190	2.8	-	-	-
ESR (<30 vs. ≥30)	0.3316	0.1340–0.8205	0.0089	2.9	-		
NLR	1.1164	1.0496- 1.1874	0.0004	24.6 *			
NLR (<4.33 vs. ≥4.33)	2.9842	1.3930–6.3927	0.0040	4.7			
PLR	1.0023	1.0007–1.0039	0.0047	12.9 *			
PLR (<304.82 vs. ≥304.82)	2.9647	1.4276–6.1567	0.0041	3.4	2.4433	1.1594–5.1489	0.0188
MLR	2.8688	1.6225–5.0726	0.0003	23.5 *			
MLR (<0.602 vs. ≥0.602)	3.4234	1.6159–7.2526	0.0017	3.8	-	-	–
SII	1.0002	1.00009–1.0004	0.0004	53.5 *			
SII (<1127.97 vs. ≥1127.97)	2.6154	1.2096–5.6546	0.0116	6.6 *	-	-	-
SIRI	1.083	1.0358–1.1312	0.0004	107.9 *			
SIRI (<2.307 vs. ≥2.307)	2.8821	1.3010–6.3843	0.0063	4.1	-	-	-
PIV	1.0002	1.00006–1.0003	0.0008	74.7 *			
PIV (<433.92 vs. ≥433.92)	3.9611	1.4981–10.473	0.0016	3.5	3.0241	1.1147–8.2040	0.0297
HALP	0.9663	0.9409–0.9924	0.0118	2.5			
HALP (<21.93 vs. ≥21.93)	2.6780	1.2173–5.8912	0.0104	3.3	-	-	-

(VIF: Variance Inflation Factor) * Data with VIF > 5 were not included in the multivariate analysis.

**Table 5:** Univariate and multivariate analyses of overall survival.

	Univariate Analysis				Multivariate Analysis		
Parameters for OS	Hazard Ratio	95% Confidence Interval	*p*	VIF	Hazard Ratio	95% Confidence Interval	*p*
Age (years)	1.0889	1.0531–1.1259	0.0001	1.9	1.0569	1.0222–1.0928	0.0011
Age (>65 years)	0.0652	0.0255–0.1665	0.0001	1.9	-	-	-
Sex (male/female)	1.4853	0.5635–3.9147	0.4136				
Treatment type (ABVD vs. BV-based)	0.0644	0.0229–0.1809	0.0001	1.7	0.1764	0.0464–0.6701	0.0108
Stage	0.5932	0.2249–1.5643	0.2781				
Liver involvement	0.9211	0.3314–2.5598	0.8740				
Spleen involvement	1.5002	0.6011–3.7441	0.3911				
ESR	1.0108	0.9972–1.0245	0.1288				
ESR (<30 vs. ≥30)	0.2510	0.0726–0.8678	0.0123	1.5	-	-	-
NLR	1.0936	1.0092–1.1851	0.0289	6.1 *			
NLR (<4.33 vs. ≥4.33)	2.9112	1.1345–7.4705	0.0228	2.9	-	-	-
PLR	1.0018	0.9998–1.0039	0.0832				
PLR (<304.82 vs. ≥304.82)	2.1376	0.8673–5.2688	0.1049				
MLR	3.0530	1.6638–5.6022	0.0003	5.7 *			
MLR (<0.602 vs. ≥0.602)	4.8429	1.9031–12.323	0.0009	2.8	-	-	-
SII	1.0001	1.00009–1.0003	0.1483				
SII (<1127.97 vs. ≥1127.97)	2.1638	0.8491–5.5140	0.0982				
SIRI	1.0697	1.0117–1.1311	0.0179	5.9 *			
SIRI (<2.307 vs. ≥2.307)	6.5192	1.8910–22.4746	0.0004	4.1	5.2829	1.4679–19.013	0.0108
PIV	1.0001	0.9999–1.0002	0.1053				
PIV (<433.92 vs. ≥433.92)	6.4814	1.4913–28.1685	0.0018	2.9	-	-	-
HALP	0.9957	0.9757–1.0160	0.6735				
HALP (<21.93 vs. ≥21.93)	1.6951	0.6671–4.3070	0.2588				

(VIF: Variance Inflation Factor) * Data with VIF > 5 were not included in the multivariate analysis.

## Data Availability

The datasets generated and/or analyzed during the current study are available from the corresponding author on reasonable request.

## Supplementary Materials

The following supporting information can be downloaded at: https://www.mdpi.com/article/10.3390/diagnostics16070980/s1, Table S1. Predictive Role of Inflammatory Parameters in Mortality Classification; Figure S1. PIV, Progression-Free Survival; Figure S2. PLR, Progression-Free Survival; Figure S3. SIRI, Overall Survival.
