# Supplementary material for: The Prognostic Significance of the Systemic Inflammation Response Index (SIRI) and HALP Score in Hodgkin’s Lymphoma
Source: Diagnostics (Basel). 2026 Mar 25;16(7):980. doi: 10.3390/diagnostics16070980 (PMC13073017; doi:10.3390/diagnostics16070980)

Table S1. Predictive Role of Inflammatory Parameters in Mortality Classification.

Parameter	AUC	%95 CI	Cut-off	Sensitivity (%)	Specificity (%)
NLR	0.585	0.491 to 0.676	≥ 4.33	63.16	63.27
PLR	0.557	0.462 to 0.649	≥ 304.82	47.37	72.45
MLR	0.669	0.576 to 0.754	≥ 0.602	57.89	74.49
SII	0.562	0.467 to 0.653	≥ 1127.97	63.16	56.12
SIRI	0.627	0.533 to 0.715	≥ 2.307	84.21	55.10
PIV	0.599	0.504 to 0.688	≥ 433.92	89.47	41.84
HALP	0.556	0.462 to 0.648	> 21.93	63.16	53.06

AUC. Area under the curve; 95% CI. Confidence interval.
Cut-off values were calculated via Youden index

Figure-S1: PIV, Progression Free Survival

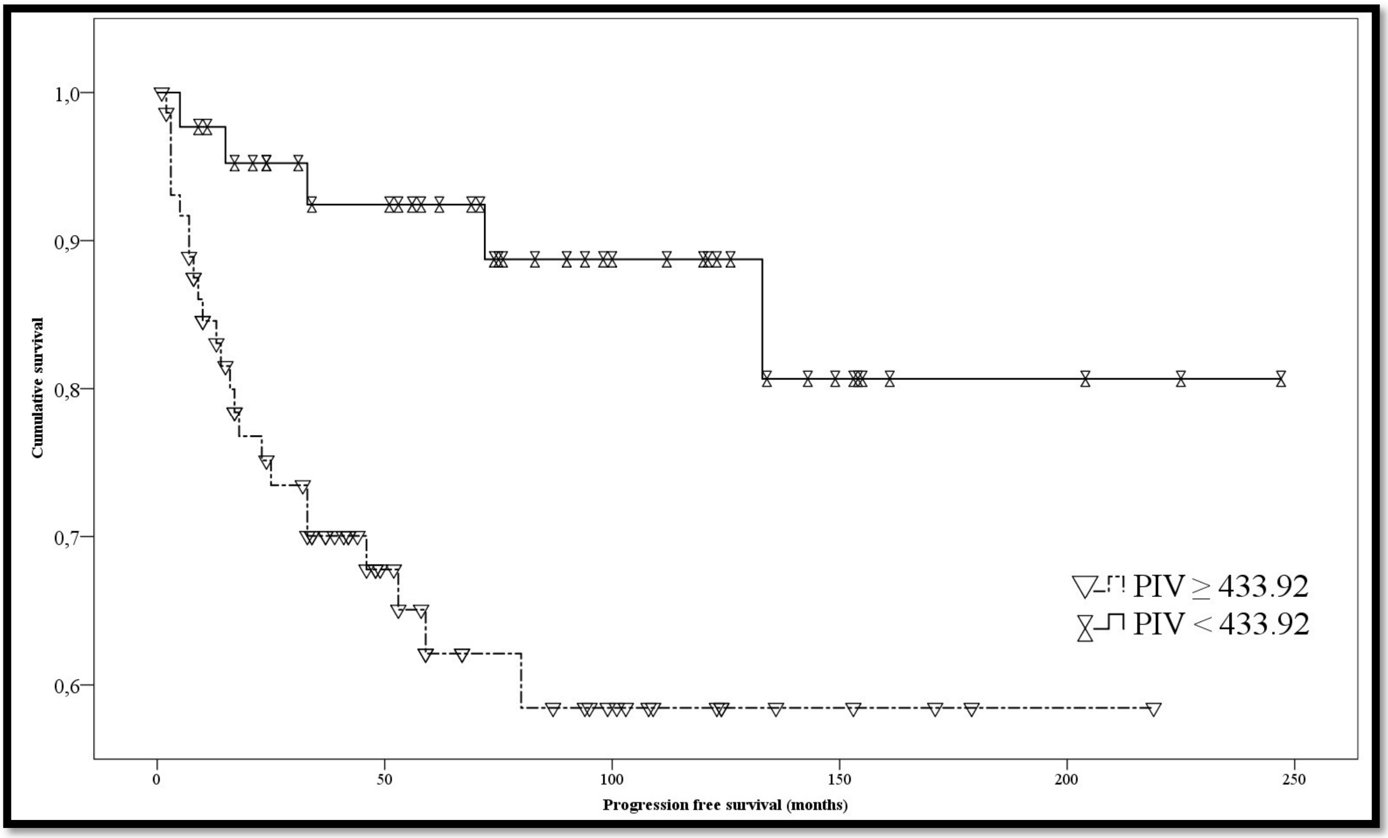

Figure-S2: PLR, Progression Free Survival

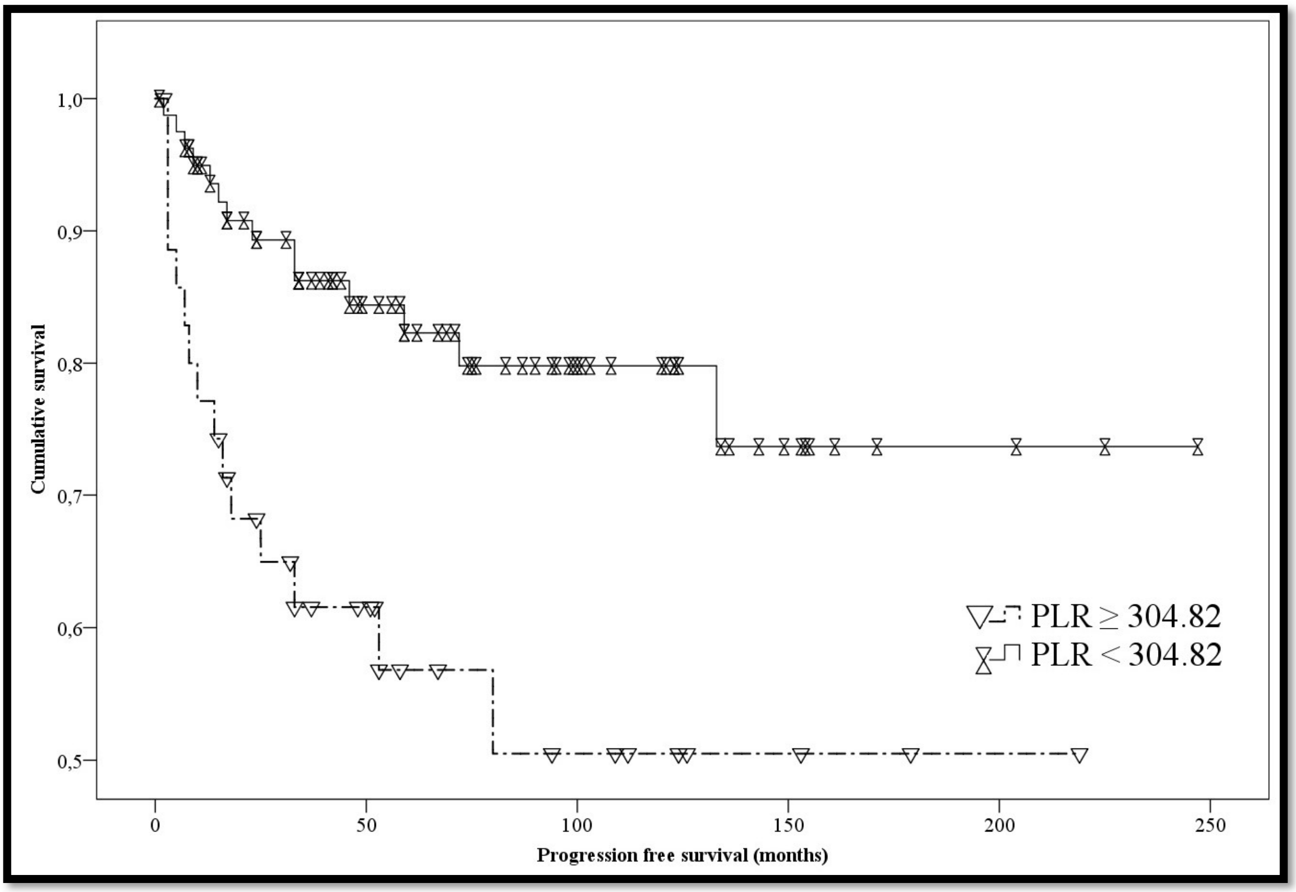

Figure-S3: SIRI, Overall Survival

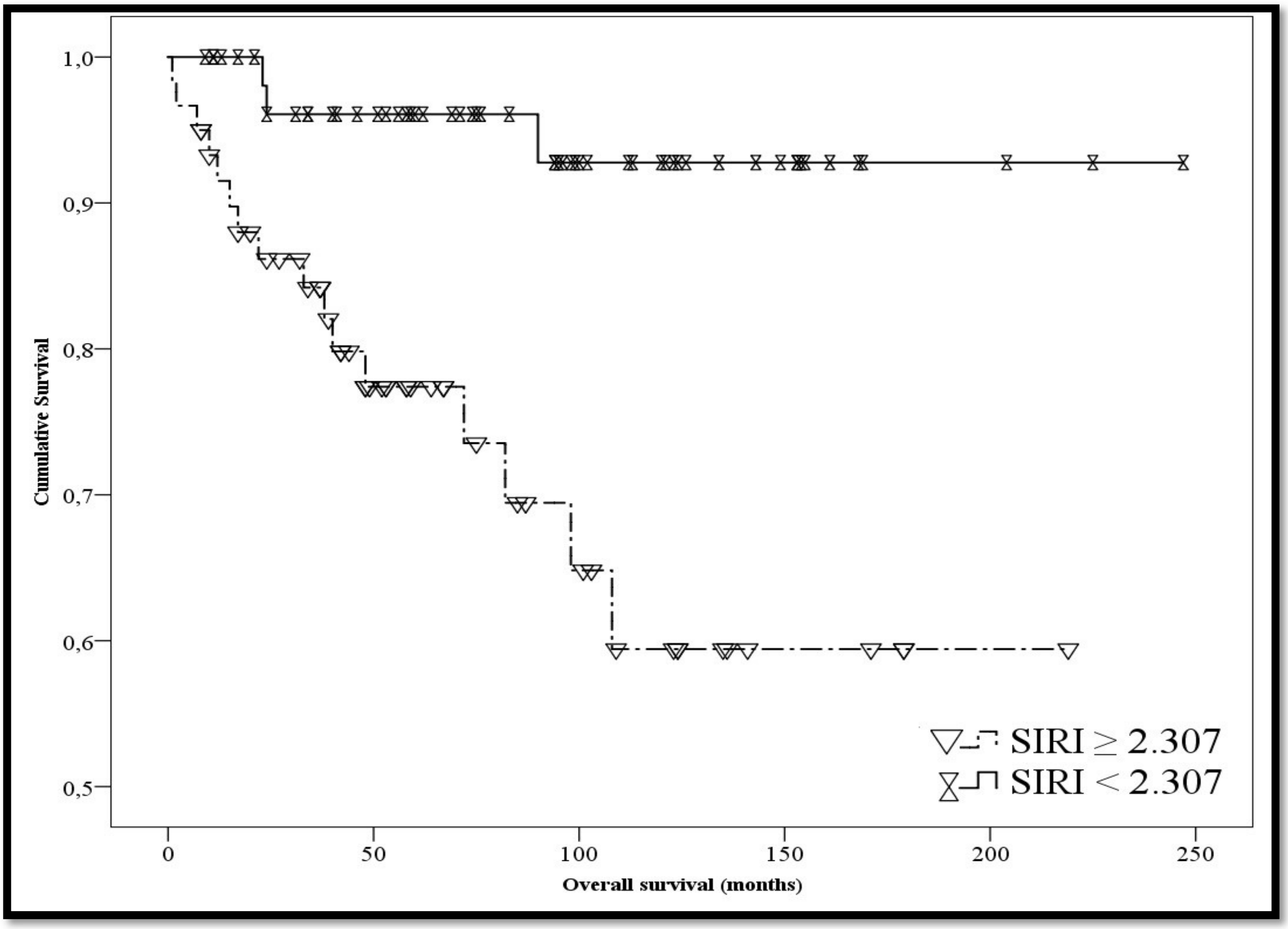

Supplement: Supplementary file 1 [file diagnostics-16-00980-s001.zip › diagnostics-4142215-supplementary.pdf]
